# StraightenUp+: Monitoring of Posture during Daily Activities for Older Persons Using Wearable Sensors

**DOI:** 10.3390/s18103409

**Published:** 2018-10-11

**Authors:** Gabriela Cajamarca, Iyubanit Rodríguez, Valeria Herskovic, Mauricio Campos, Juan Carlos Riofrío

**Affiliations:** 1 Department of Computer Science, Pontificia Universidad Católica de Chile, Santiago 7820436, Chile; mgcajamarca@uc.cl (G.C.); iyubanit@uc.cl (I.R.); 2 School of Medicine, Pontificia Universidad Católica de Chile, Santiago 8331150, Chile; macampos@med.puc.cl; 3 Department of Computer Engineering, Universidad de Santiago de Chile, Santiago 9170124, Chile; juan.riofrio@usach.cl

**Keywords:** wearable, user experience, older users

## Abstract

Monitoring the posture of older persons using portable sensors while they carry out daily activities can facilitate the process of generating indicators with which to evaluate their health and quality of life. The majority of current research into such sensors focuses primarily on their functionality and accuracy, and minimal effort is dedicated to understanding the experience of older persons who interact with the devices. This study proposes a wearable device to identify the bodily postures of older persons, while also looking into the perceptions of the users. For the purposes of this study, thirty independent and semi-independent older persons undertook eight different types of physical activity, including: walking, raising arms, lowering arms, leaning forward, sitting, sitting upright, transitioning from standing to sitting, and transitioning from sitting to standing. The data was classified offline, achieving an accuracy of 93.5%, while overall device user perception was positive. Participants rated the usability of the device, in addition to their overall user experience, highly.

## 1. Introduction

The level of activity associated with movement among older persons can be a determinant factor in their general health and functional state. Evidence suggests that factors such as a low level of physical activity [1] and sagittal imbalance [2] contribute to the deterioration of health and quality of life. The monitoring of human posture and analysis of movement during daily activities can be useful to remotely control the health of patients, especially older persons with limited mobility and increased dependence [3]. In the long term, this monitoring could be used to identify the behaviour, form and intensity with which activities are carried out [4]. This information may be of use to doctors and researchers who are seeking to understand the development and progression of an illness, since certain chronic conditions could be related to time spent in inappropriate postures or general inactivity.

There are several approaches to measure human posture and movement which vary according to specific needs, viability and accuracy. Subjective methods such as diaries, questionnaires and surveys are low cost, but may be affected by recall bias or require interpretation [5]. Observation-based methods, such as video-recording, have the benefits of direct observation, but are difficult to implement for large groups [6] or people who are not in a fixed location. Another approach is to use sensors—either smartphone sensors [7], smartwatch sensors [8], or fixed sensors attached to the body, which have been used to measure physical and physiological parameters; e.g., measuring metabolic energy consumption [9] and bodily posture stability [10,11,12]; predicting falls [13,14,15]; and detecting regular daily activities [16,17]. Sensor-based physical activity classification systems can be described in terms of several factors: dataset (where the activities were collected and which activities were chosen as relevant), number of sensors, placement of sensors, features set, window size and classifier [18]. The high variety among studies (in device type, placement and data interpretation) presents a challenge when comparing results between studies, and more research is needed to establish guidelines that make it possible to compare accelerometer data for older users [5].

There is a large body of research on accelerometer-based devices to monitor daily activities. A systematic review of accelerometer-based activity monitoring research up to 2010 found that, although a higher number of accelerometers provide increased precision, a single waist-mounted accelerometer could be a good compromise between comfort and accuracy [19]. Several recent studies have proven this to be true. One accelerometer has been successfully used to detect some activities (e.g., walking, jumping, running, being stationary, and transitions between sitting and standing, and kneeling and standing) [20]. One wrist-based accelerometer was used to discriminate between walking and other activities [21], and one accelerometer placed on the lower trunk was able to distinguish walking and stair ascent/descent [22].

Some studies indicate that portable sensors placed at the waist provide the highest accuracy to predict body movements [23], since a person’s centre of gravity is closer to the waist and, therefore, the generated data may be more reliable [24]. Force sensors along with acceleration sensors have also been placed on the plantar surface of the foot to measure information for reliable recognition of postures and typical daily activities [25,26]. The position of the sensors must balance unobtrusiveness and accuracy [27]. The placement of accelerometers in multiple locations can be annoying for the user, especially in long-term monitoring applications [28]. Placing accelerometers at the waist or hip have caused compliance issues, with participants citing discomfort and inconvenience of wearing the device for long periods [29]. Additionally, placing accelerometers at the waist requires consideration for participants with obesity [30], with some studies limiting the amount of time users wear devices for this reason [31]. A recent study found the wrist to be the preferred placement of a wearable, followed by the chest, and finally, the waist [32]. Another study found that older users showed no clear preference over placement of a wearable on the arm, neck, waist or wrist [33].

Wearable flexible sensors, e.g., flexible and stretchable strain sensors, have been used to monitor the movement of users’ bodies [34]. These sensors have also been used to monitor posture, e.g., by positioning sensors at the knee and the hip, researchers were able to classify standing, sitting, sitting with extended knees and supine poses [35]. The challenges regarding these sensors are similar to those of accelerometer-based wearables, e.g., large amounts of data are generated, and users’ comfort (and the effect of the sensors on the body) must be taken into account [34].

The movement patterns of young, healthy individuals may differ from older people or people with mobility issues. For instance, gait disorders, slow walking, and using a walking frame increase step tracker errors [6]. Several studies have focused e.g., on people with gait abnormalities and neuromuscular disorders [36] or stroke patients [37]. Other studies have focused on detecting activities of older participants. Two accelerometers (placed on the trunk and thigh) were used to classify six activities of daily living for elders, with a 2.8% misdetection rate [38]. Another study specifically focused on hand gestures (e.g., eating, drinking, brushing hair) [27].

Although a large body of work has studied the number, placement, and classification algorithms for physical activity detection, there has been little focus on user experience, i.e., how older users relate to this type of wearables and how to improve their experience with them. Recent work has highlighted this need, e.g., evaluating a sensor-based fall risk assessment belt with a user experience perspective [39]. When designing wearable technology for older persons, it is important to consider that older persons are a highly heterogeneous group [40] and that they engage with the internet and technology in a different way to younger persons. Older users are faced with unequal access and lower digital skills barriers to adopt and use chronic disease monitoring devices [41]. There are several challenges when introducing wearables to older people: possible physical and neuro-degenerative limitations must be taken into account [42], and older users have safety concerns and fear that this type of technology will increase isolation [43]. The design of technology for use by older persons should consider the conditions of users, the effects of aging, cultural context and common chronic illnesses in order to ensure that this segment of society does not become isolated due to its inability to use a mobile telephone, access the internet or understand the latest interfaces [44].

The main objective of this paper is to present an evaluation of the user experience of older persons who interact with a low-cost wearable device to monitor daily activities by means of spinal posture. Designing, implementing, and testing wearables with this population, and understanding their needs, constraints, and expectations, contributes to the growing body of knowledge on how elderly users interact with new types of technology. The proposed device features three accelerometers/gyroscopes distributed across a harness that is attached to the back. The article is structured in the following manner. Section 2 describes the implemented device, called StraightenUp+. Then, Section 3 describes the materials and methods used in the evaluation of the device. Section 4 presents the results of the experiment conducted on the proposed activity recognition system. Section 5 discusses the results, and Section 6 presents our conclusions and research limitations.

## 2. StraightenUp+: A Wearable Device to Monitor Posture for Older Users

This section describes the design and development of the StraightenUp+ device, which was an iteration of a previous prototype called StraightenUp. We describe the first prototype, and its evolution into StraightenUp+, along with implementation details.

### 2.1. StraightenUp: A First Prototype for Static Postures

**Figure 1:**
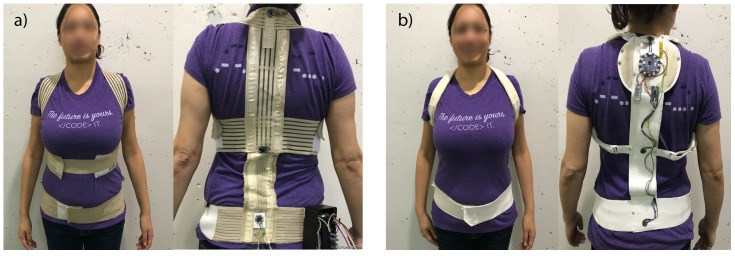
StraightenUp design: (**a**) phase I; (**b**) phase II.

First, we developed StraightenUp, an initial prototype of a wearable device to statically measure the inclination of the spine. It consisted of three accelerometers in a harness as well as a box, stuck to the side of the harness, in which some Arduino components were encased. The device is shown in Figure 1a.

We used the AttrakDiff questionnaire [45], the StraightenUp prototype and semi-structured interviews, to evaluate the usability, functionality and design of this device. The evaluation was performed by 30 higher education students (7 women and 23 men), of which 14 reported to experience back pain. Participants used the StraightenUp prototype and performed six different bodily postures. Each posture lasted approximately 20 s (50 measurements were recorded during this period).

To assess whether the device could be used to broadly measure spine inclination, a classification model based on a decision tree was used. The classification model was able to accurately distinguish between six static bodily postures; this represents 99.5% of cases correctly identified. However, it is important to note that these six body postures were artificial and several were purposefully distinct, in order to achieve a preliminary validation of the device. Data from the sensor located in the upper part of the torso showed greater dispersion in some postures, probably due to the position of the head, while the sensor located on the mid-section of the torso had heterogeneous data with regard to data from the other two sensors. This distribution may be due to the presence of atypical data caused by the physique of particular participants.

Regarding user experience, StraightenUp was perceived as an unpresentable device, i.e., it lacked the appearance of a finished product. Even though participants thought the device could be useful, they raised several user experience issues: they thought the device was difficult to put on and felt that they needed assistance to adjust it correctly because the device had three independently adjustable straps. They also disliked the size of the box on the side of the device. A complete description of the device and experiment may be found in [46].

### 2.2. StraightenUp+ Design Rationale and Functional Requirements

After the first experience regarding user experience with a wearable to monitor posture, we devised four design requirements for the next version of the prototype, StraightenUp+.

First, the device should be usable with *minimal adjustment*. The first version of StraightenUp had several adjustable straps, which users were nervous about manipulating. Second, the device should be *easy to put on and take off*, especially considering the physical constraints of older users. This is important for allowing older users to choose when they want to wear the device and feel, in this regard, independent. Third, the device would need to be *self-contained*, that is, not require additional components (such as the box, stuck to the side of the harness, present in the first version). The reason for this is that it is difficult for end users to understand the need for such a box, and, especially for older users, the device should be easy to understand. Finally, the number of straps, especially those over the stomach, should be as few as possible, to *accommodate different body shapes* and not make users uncomfortable.

Regarding functionality, StraightenUp only measured spine posture in static positions. The new version of StraightenUp+ would have to allow users to walk and perform daily activities without restriction.

### 2.3. StraightenUp+: A Wearable for Older Users

We addressed the aforementioned design and user experience problems, and created a new StraightenUp prototype called StraightenUp+. The design considerations were incorporated in the following way: (1) In contrast to the previous design that had three adjustable straps that crossed on the upper, mid and lower torso (see Figure 1a), we chose a modified harness vest in the form of a backpack with two straps that pass over the shoulders and a further strap around the waist (see Figure 1b). The harness is only adjustable at the waist; (2) The device is shaped like a backpack, so users only need to insert their arms and adjust the waist strap in the front of the device, still ensuring the secure positioning of the sensors. This design is less confusing and easier to put on and take off; (3) In contrast to the first design, which had a box located on the lower right side of the device (see Figure 1a), the sensors and main card located across the rear strap (see Figure 1b) and could be easily covered with some fabric; (4) The strap that made users uncomfortable, as it stretched over the stomach, was removed.

The first version of the device had three accelerometers which were only tested with users who were in still, predefined positions. To improve accuracy and the ability of the prototype to measure posture while users did activities while moving (such as walking or sitting), three inertial sensors were used, each one composed of a triaxial accelerometer and a triaxial gyroscope.

### 2.4. StraightenUp+: Implementation

#### 2.4.1. Hardware Components

**Figure 2:**
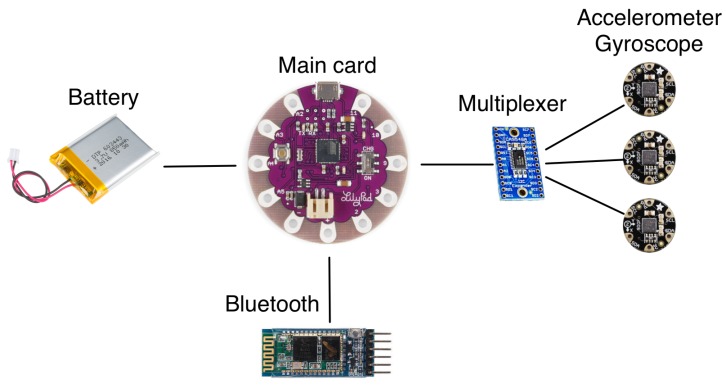
General architecture of the StraightenUp system.

The device is based on a Lilypad Arduino ATmega32U4 main card (Atmel, San Jose, CA, USA) with three LSM9DS0 FLORA 9-DOF Accelerometer/Gyroscope/Magnetometer (STMicroelectronics, Generva, Switzerland) connected to a TCA9548A 1-to-8 I2C multiplexer (Texas Instruments, Dallas, TX, USA), and a 3.7 V Lithium-Ion 1 Ah battery. The components are shown in Figure 2.

#### 2.4.2. Communication Protocols

The accelerometer data is captured and transferred through Bluetooth for processing. The Bluetooth protocol was designed for short range communication and low battery consumption [47], and can be used e.g., to connect to a nearby mobile phone carried by the user to transfer the captured data, similarly to other wearable devices (e.g., Fitbit). To be able to remotely monitor the user’s activities, the mobile phone would have to upload the data to a defined server. For this particular implementation and experiment, the data was transferred via Bluetooth to a nearby laptop, for its posterior analysis.

#### 2.4.3. Trunk Posture Measurement

The three inertial sensor modules (accelerometers and gyroscopes) were located on the back of the subjects, in the upper, middle and lower trunk, with elastic bands crossing at the shoulders and a band around the waist (see Figure 1b) to measure the orientation of the upper body. Just like any inertial instruments, the accelerometer/gyroscopes needed to be calibrated before being used for the first time. The calibration was defined as the process of comparing instrument outputs with known reference information [48]. In this sense, we first placed the sensors horizontally with the *z*-axis faced down to reduce errors of sensitivity and offset from the raw measurements [49,50,51]. Then, the orientation of the sensor was calculated by combining calibrated signals from accelerometers and gyroscopes [52]. Finally, we used a complementary filter to obtain a measurement of the complete and precise orientation in relation to the direction of gravity and the magnetic field of the earth. We decided to use a complementary filter, based on the pre-filter proposed in [53], due to its acceptable level of accuracy and low computational load. All of this preprocessing was done in the Arduino main board.

## 3. Materials and Methods

In this section, we describe the materials and methods used to evaluate StraightenUp+.

### 3.1. Study Context

This study was conducted in a residential home for older persons in Santiago, Chile. In Chile, more than 50% of older persons experience health problems related to pain in the back, knees, hips or joints. One in ten claims to have experienced a fall [54]. A total of 1.56% of older persons live in residential homes; institutions that are admitting a rising rate of residents of advanced age (7.6%, ≥90 years old), and in which women (61%) and single persons (34%) are particularly numerous. Among older persons with disabilities, 15% of individuals who experience physical impairment or paralysis of some kind, and 13% of those classified as having mental disabilities, live in residential institutions [55]. Furthermore, older persons who are already institutionalized experience greater loss of function due to inactivity [56], isolation becomes more severe [57] and depression rates are high.

According to information provided by the institution where the study was conducted, 33% of its residents are independent and the rest are semi-independent. Over 50% of residents use a device for mobility purposes. In Chile, 17.4% of adults report having no prior experience with computers, and 52.4% of adults have a score equal to or lower than level 1 in problem solving in technology-rich environments [58]. However, the digital skills of those older adults residing in the institution were lower. We conducted a preliminary observation of 69 subjects (31 men and 38 women) at the residential home, while they carried out their daily activities, to understand the context, characteristics, conditions and limitations of our potential users. Based on these observations, we generated a list of the postures to be evaluated during the experiment.

### 3.2. Collected Information

The evaluation was done during October 2017. The following six types of information were collected:

- DIGCOMP: DIGCOMP is a questionnaire used to assess four areas of digital competences: information, content creation, communication and problem solving. Each user is categorized into one of four possible groups: no, low, basic, or above basic [59].
- AttrakDiff: AttrakDiff is a questionnaire used to measure hedonic and pragmatic qualities of a device and it allows users to rate the usability and design of a product. It uses a scale of −3 to 3 (0 represents neutrality). The AttrakDiff questionnaire consists of four dimensions: pragmatic quality, hedonic quality identity (HQ-I), hedonic quality stimulation (HQ-S) and attraction [45,60].
- Frail Elderly Functional Assessment (FEFA) questionnaire: FEFA consists of 19 items and assesses function among frail older persons at a very low activity level [61].
- Logged data: The StraightenUp+ prototype collected continuous information from the three inertial measurement units of 9 degrees of freedom of sensors (incorporated accelerometer and gyroscope). The recorded data relates to the inclination in the *x*- and *y*- axes. For our purposes of this study, the *y*-axis captures lateral movement (from left to right), while the *x*-axis captures horizontal movement (forward and backward).
- Observation data: One researcher observed the participants, noting the times in which they performed each posture in an audio recording, as well as any additional problem or issue that arose during the experiment.
- Interview data: A semi-structured interview was conducted to understand participant comfort, motive and frequency of use in relation to the device. Each interview was recorded (audio), transcribed and assigned a code (P1 to P30). Users were asked about the comfort of the device, whether they would use it, when and for how long, and what they liked and disliked about the device.

### 3.3. Participants

Our participants were 30 older persons (15 women and 15 men) aged between 60 and 83 years (average age: 77.8; standard deviation: 6.13) and living in a residential home. Regarding their digital skills, 28 participants have none and 2 have high or medium skill levels. The inclusion criteria were as follows: over 60 years old, and an absence of moderate or severe cognitive problems. According to the FEFA scale, on a scale from 0 to 55, participants had an average score of 24.3, with a minimum score of 15 and a maximum score of 38, denoting some functional deterioration. Each participant read and then signed the informed consent form.

### 3.4. Procedure

**Figure 3:**
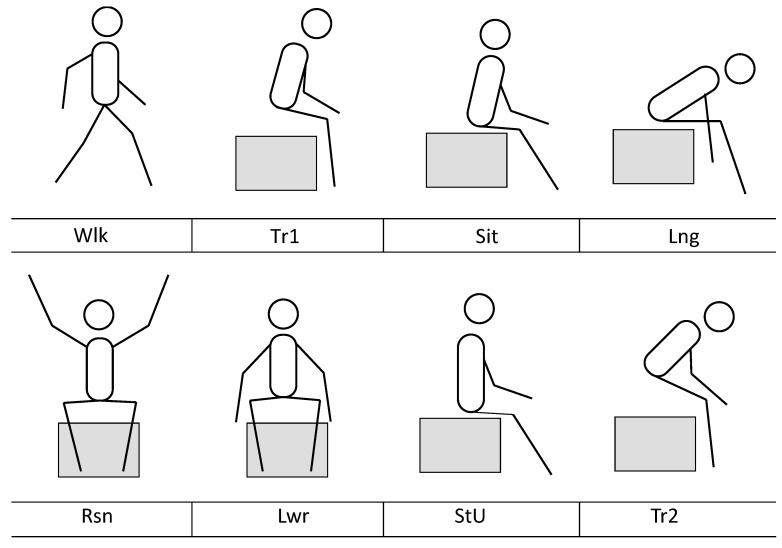
All eight classified postures.

The experiment lasted between 50 and 65 min per participant, and two researchers participated in each complete experience. At the start of the experiment, one researcher gave a brief introduction about the purpose of the investigation, answered relevant questions, and the participant then signed the informed consent form (10 to 15 min). The participant was then asked to put on the device and perform eight activities in a predefined order: walking (Wlk); standing to sitting transition (Tr1); sitting (Sit), leaning forward (Lng); raising arms (Rsn); Lowering arms (Lwr); sitting upright (StU) and sitting to standing transition (Tr2) (see Figure 3). While the participant performed these activities, one researcher observed and recorded notes in audio, in order to record each transition between activities. The data that was captured by the device was transferred via Bluetooth to the researchers’ laptop computer, which was nearby. After completing the activities, participants completed the DIGCOMP, AttrakDiff, and FEFA questionnaires (8 min). Finally, the researchers performed a semi-structured interview, asking questions about the experience of using the device (8 min). After the experiment, the captured data was labeled offline, using the audio notes, to mark each transition and have a labeled data set to work with.

## 4. Results

This section outlines the results of the tests undertaken into the accuracy of sensors to measure postures, and describes aspects about the user experience. First, to create a labeled dataset, two researchers labeled the data from each sensor and participant with the posture the participant was performing, obtained from the recorded audio notes. Second, to analyse the accuracy of the sensors regarding statistical descriptions, we used the *R* software (version 3.5.1, R Foundation, Vienna, Austria) [62]. Then, we used algorithms based on a decision tree using the Weka software (version 3.8, The University of Waikato, Waikato, New Zealand) [63] for activity classification. The usability and appearance of the system were evaluated via the AttrakDiff questionnaire. Finally, to evaluate qualitative characteristics, two researchers codified interview notes and used thematic analysis to identify and analyse emerging themes [64].

### 4.1. Descriptive Statistics

**Figure 4:**
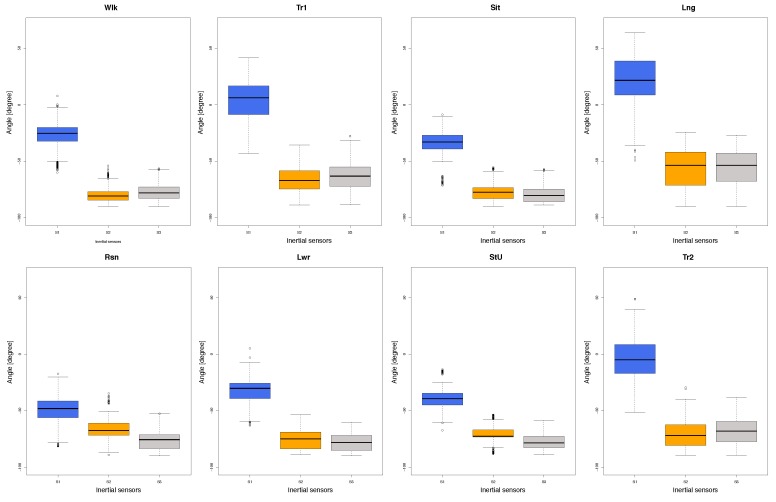
Distribution of data on the *x*-axis for each sensor during the monitoring of torsos while residents carry out specific activities.

The graph in Figure 4 displays the distribution of data on the *x*-axis for each sensor and each posture. It is possible to see a difference between sensor 1 (s1, located in the upper part of the torso), and sensors 2 and 3 (s2 and s3, located in the mid- and lower part of the torso respectively), which are mostly similar. In line with the monitored activities, during the leaning forward (Lng) posture, the three sensors record data that varies markedly in comparison to the other activities. It is possible that this particular posture requires greater effort from the participants, the majority of which have a below average physical condition. Conversely, the data collected by the three sensors when the participant is sitting is homogeneous and shows minimal variation.

### 4.2. Posture Classification

We classified the labeled data by using a J48 decision tree in Weka. The postures were recognized with an accuracy rate of 93.5% for the test cases used in this study. The confusion matrix in Table 1 shows that the majority of confusion was introduced by the Walking activity. The eight states of movement include a time period of upright posture in which the three sensors tend to align at approximately 80 degrees, although this value depends on the spinal structure of each individual participant. However, the activity that generated the least amount of confusion in relation to walking was *raising arms*, since this activity requires more backward movement than upright positioning.

Having analysed the distribution of data (see Figure 4) and the confusion matrix (see Table 1), we can observe that sensor one (s1) may be the best at predicting movement detection. To explore this aspect further, we decided to compare posture classification according to the number of sensors used (see Table 2). For this, we classified the data using information from one of the three sensors or any combinations of them. The results show that the best classification is achieved by all three sensors, although, when using any two sensors, the data is classified correctly at about 90% of instances.

### 4.3. Experience with StraightenUp+

**Figure 5:**
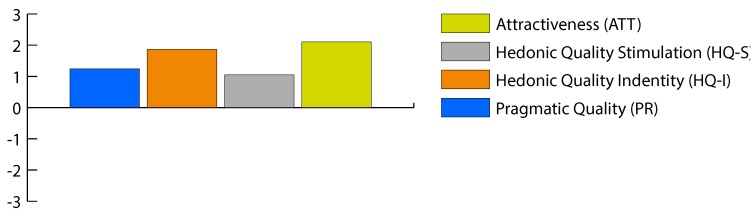
Average values for pragmatic quality (PQ), hedonic quality identification (HQ-I), hedonic quality stimulation (HQ-S), and attractiveness (ATT).

The AttrakDiff scores (which are on a scale of −3 to 3) were positive for all 30 participants, and greater than or equal to 1.5 for 18 participants (60%), i.e., general user experience of StraightenUp+ was classified as good. The highest scoring characteristic was the attractiveness quality which scored 2.11, followed by hedonic identity with a score of 1.87. Then came the pragmatic quality characteristic, which scored 1.24. Finally, the lowest scoring quality was hedonic identify stimulation with a score of 1.05. The average values recorded are shown in Figure 5. Consequently, users view the device as having a good appearance, they are able to identify with it, and they find it both stimulating and motivating. Participants classified the device in a highly positive way in terms of prospective use (average scores for attractive, manageable, presentable, and creative were 2.3, 2.6, 2.8, and 2.6, respectively). However, the technical-human concept was classified as primarily a technical one (−2.3 average score), and likewise the demanding-non-demanding concept received negative scores (−0.8 average score), i.e., StraightenUp+ was classified as a non-demanding device, the use of which requires no prior knowledge.

### 4.4. Interviews

The main themes that arose from the thematic analysis include: perception of device, motivation for use, frequency of use and expectations. Each is described below. Quotes from participants are provided (translated from Spanish), and participants are numbered P1 to P30. Some comments were spontaneously given by the participants; therefore, although the number of comments are specified, this does not mean that the rest of the participants necessarily disagree with the comment.

1. **Perception of device:** Twenty-eight of the participants felt that StraightenUp is comfortable, and two felt that it was uncomfortable. Furthermore, four participants commented that the device felt imperceptible, and one that the straps made it adaptable to different bodily physiques. *“I think it’s comfortable, it doesn’t bother me at all when I move, the straps don’t bother me one bit”* (P4). One participant expressed their perception of the device using a comparison: *“It was normal, I didn’t notice it, it was like a regular backpack”* (P25). One participant indicated the need to include instructions or indicators to improve user experience in putting it on and taking it off. *“You could improve it to make it so that I can’t make a mistake, something that prevents mistakes when putting it on”* (P18).
2. **Motivation for use:** Participants valued the device as an article that can have a positive health impact. Five participants stated that it could prevent them from adopting bad postures, three participants valued that it could help them understand or diagnose their posture, and two participants that it could improve their health. One participant views the device as a support tool. *“When I’m walking, it (the device) helps me to stay on my feet and I feel like I’m walking okay. I go out a lot, I’m 84 years old, I take the bus, I take the subway, and I would like to be sure that I’m not going to fall over or that I won’t be knocked down”* (P24).
3. **Frequency of use:** Fourteen participants thought the best moment to use StraightenUp+ is during physical activities, e.g., walking and exercising. *“I mean, during more physical activities, like running, walking, biking during the morning”* (P10). Conversely, three participants claim the device is best used during periods of rest, when the user cannot be seen or is largely inactive. *“Well, it’s complicated because the jokes would start, the negative comments and all that, and here it’s difficult, so I would say during the two hours dedicated to rest”* (P7).
4. **Expectations:** Sixteen participants would like to use StraightenUp+ permanently to gain greater understanding of the system and to become more accustomed to how it works. *“I’ve never seen anything like it, it helps you to ask yourself things and then they become second nature… I’ve never used it, I asked how I was going to get around with this thing on, if I used it at all that is, everything you get used to using… I have got used to using so many things over the years but I never thought I’d use this, but if it can help you, you should use it”* (P23). Ten participants would use it when required or when they felt pain or had a health problem. Finally, four participants were not interested in using the device at all, or unsure about wanting to use it. *“I wouldn’t use it, for one. I’m not used to it, and second, it would mean being constantly concerned with walking perfectly, like a machine”* (P1). Furthermore, three participants commented that the device should be used under the clothes rather than over them. *“It should be used under your clothes because nobody would notice, nobody would criticise me, nobody would be pointing out that I use it or not”* (P4).

## 5. Discussion

This paper has described and evaluated a harness vest-shaped device for older people, in which three accelerometers are placed on the upper, mid and lower torso. Performance evaluation of the device showed fairly accurate classification, with a rate of incorrect detection of 6.5%. A similar recent study in elders had a rate of misdetection of 2.8% [38]—however, in that study, participants used sensors in the trunk and thigh, and the system classified six activities, while our work classifies eight. The classification error in our study may be partly caused by the complex body movement of the elderly participants due to their functional deterioration. The highest misclassification rate was the sitting to standing transition (Tr2, 59.8%). This is probably due to the complex dynamic movement of the trunk to perform this action. In general, the elderly participants with low functional capacity stand with a greater flexion of the upper body [65,66], and the choice of standing strategy is related to the reduction of muscle strength [67]. In this study, we observed that the majority of residents with functional impairment used support such as handrails, a cane or a walker to perform the movement from sitting to standing. Previous work has shown that gait disorders, slow walking, and the use of walkers increase tracking errors [6]—so it may be easier to classify older people’s daily activities when their functional deterioration is lower. The transitions are more difficult to classify as they are short, but at the same time, have a high variability (in time and type of movement) between participants with varying degrees of mobility. It should be recognized that this study did not consider free activities, since residents were asked to carry out a sequence of activities that were explained previously. For this reason, future experiments should use protocols of less controlled activity (e.g., [68]). In addition, the performance of our device could possibly be further improved by incorporating additional filters (e.g., [53]).

Previous studies on remote monitoring devices for older users have placed sensors on the trunk, sacrum, abdomen, chest, wrist, pocket or neck, with some placing sensors on several locations (e.g., chest, wrist, thigh and ankle) [6]. Although, e.g., the wrist has been found to be preferred by young users [69], older users have not declared a clear preference [33], and in our observations, in people using support devices, the wrist often remains stationary. Our device was placed on the back of the users, with users finding it to be comfortable and feeling it provided some type of support.

During evaluation of system performance in the natural assisted-living setting, interviews and questionnaires were conducted on aspects related to design and user experience. Residents valued the device fairly highly, highlighting its comfort, practicality and familiarity. They compared the device to an item of clothing, likening it to an imperceptible vest or backpack. These results may be related to a lack of direct experience in the use of portable technology [70] and individual and social influence [71].

Many participants of this study declared to have never used a computer or the internet, and some only use analogue telephones to make or receive calls. However, they received the device in a positive manner. One possible reason for this is that StraightenUp+ requires no input or interaction from users besides putting the device on, which is one of the aspects of the device that they commented on. This contrasts with previous findings in which users wanted visualizations of their own data and behavior prompts [39], possibly because of the lower digital skills of our participants.

The way of life varies between older persons who live in a residential home and people who reside in their own homes [72]. For older adults in institutions, the decline in physical and mental health, isolation, and loss of function prevent active aging [73]. Furthermore, higher levels of activity have been correlated with lower levels of depression in institutionalized and community-dwelling older people [74]. For these reasons, monitoring physical activity and taking actions to promote activity is especially important in institutionalized older adults.

## 6. Conclusions

We have presented a device to monitor bodily posture using three sensors attached to the torso. The development, design and evaluation of the system were undertaken in two stages: (1) developing a posture monitoring device that takes account of technological factors as well as user experience factors; and (2) understanding attitudes and perceptions of older persons in residential homes towards the monitoring of bodily posture using a portable device attached to the torso. Participant experience with the device was highly positive and they highlighted its comfort, usability and familiarity (saying it was like putting on an item of clothing). In general, the evaluation results are relevant since they demonstrate that this type of device is well accepted by older persons with low digital skills and functional deterioration in residential homes; that it is considered attractive, easy to use and produces a sense of satisfaction among users; and that its use requires no advanced technological knowledge. However, it was identified that the addition of a tactile or auditive instruction component was needed to improve user experience in putting the device on and taking it off. These results can be incorporated into the design of future posture monitoring systems and may be used for more extensive tests, over more prolonged periods of time and among a group of older persons who live in an independent manner. This is because our research includes several limitations. These include older person participants not using our system over a long period of time; having limited digital competences; showing moderate functional deterioration; and living in just one particular private residential care institution in Santiago, Chile.

## Figures and Tables

**Table 1:** Confusion matrix for activities.

	Wlk	Tr1	Lng	Sit	Rsn	Lwr	StU	Tr2	TP Rate %	Precision %
Wlk	3158	6	25	6	4	3	4	3	98.4%	93.1%
Tr1	43	289	2	41	1	0	2	9	74.7%	85.8%
Lng	60	2	772	0	10	5	3	0	90.6%	93.1%
Sit	34	12	5	1213	2	2	1	4	95.3%	94.0%
Rsn	12	3	14	1	709	9	16	0	92.8%	95.1%
Lwr	18	1	4	5	7	443	4	1	91.7%	96.4%
StU	18	1	5	2	9	2	882	0	96%	90.3%
Tr2	49	23	2	23	5	2	3	159	59.8%	93.5%

**Table 2:** Accuracy according to the number of sensors used (X means that the corresponding sensor was considered in the classification).

S1	S2	S3	Correctly Classified Instances %
X	-	-	73.7 %
-	X	-	76.6 %
-	-	X	76.8 %
X	X	-	90.1 %
-	X	X	90.1 %
X	-	X	90.4 %
X	X	X	93.5 %
